# Ecological Factors and Adolescent Marijuana Use: Results of a Prospective Study in Santiago, Chile

**DOI:** 10.3390/ijerph110303443

**Published:** 2014-03-21

**Authors:** Jorge Delva, Wonhyung Lee, Ninive Sanchez, Fernando H. Andrade, Andrew Grogan-Kaylor, Guillermo Sanhueza, Michelle Ho

**Affiliations:** 1 School of Social Work, University of Michigan, 1080 S. University, Ann Arbor, MI 48109, USA; E-Mails: ninive@umich.edu (N.S.); agrogan@umich.edu (A.G.-K.); 2 Urban and Regional Planning, University of Michigan, 2000 Bonisteel Blvd., Ann Arbor, MI 48109, USA; E-Mail: elsalee@umich.edu; 3 University of Michigan Substance Abuse Research Center, 1080 S. University, Ann Arbor, MI 48109, USA; E-Mail: fandrade@umich.edu; 4 School of Social Work, Pontificial Catholic University of Chile, Avenida Vicuña Mackenna 4860, Santiago, Chile; E-Mail: gesanhue@uc.cl; 5 School of Engineering and Applied Science, University of Pennsylvania, 46035 White Pines Dr., Novi, MI 48374, USA. E-Mail: michelleho86@gmail.com

**Keywords:** marijuana use, adolescents, neighborhood characteristics, poverty, crime, drug problems, systematic neighborhood observations, Chile

## Abstract

*Purpose*: Despite the growing evidence that ecological factors contribute to substance use, the relationship of ecological factors and illicit drugs such as marijuana use is not well understood, particularly among adolescents in Latin America. Guided by social disorganization and social stress theories, we prospectively examined the association of disaggregated neighborhood characteristics with marijuana use among adolescents in Santiago, Chile, and tested if these relationships varied by sex. *Methods*: Data for this study are from 725 community-dwelling adolescents participating in the Santiago Longitudinal Study, a study of substance using behaviors among urban adolescents in Santiago, Chile. Adolescents completed a two-hour interviewer administered questionnaire with questions about drug use and factors related to drug using behaviors. *Results*: As the neighborhood levels of drug availability at baseline increased, but not crime or noxious environment, adolescents had higher odds of occasions of marijuana use at follow up, approximately 2 years later (odds ratio [OR] = 1.39; 95% CI = 1.16–1.66), even after controlling for the study’s covariates. No interactions by sex were significant. Discussion: The findings suggest that “poverty”, “crime”, and “drug problems” may not be synonyms and thus can be understood discretely. As Latin American countries re-examine their drug policies, especially those concerning decriminalizing marijuana use, the findings suggest that attempts to reduce adolescent marijuana use in disadvantaged neighborhoods may do best if efforts are concentrated on specific features of the “substance abuse environment”.

## 1. Introduction

Cities in Latin America have experienced considerable physical and socioeconomic (SES) changes in the past two decades [1]. Such changes can create an actual disadvantage or a sense of relative disadvantage in the neighborhood environment and among residents. For example, in Santiago, Chile, where the present study was conducted, urbanization and new types of development (e.g., gated communities and high-rise buildings) have created disparities in housing and tensions between groups of different SES with a corresponding rise in social disorder [1]. In Chile, there tends to be greater fear of crime in urban areas [2] and Santiago residents living in low-income communities identify delinquency (including the selling of illicit drugs) as a primary and significant public health concern in their daily lives [3,4]. 

Considering the multiple environmental changes in urban areas, the purpose of this paper is to disaggregate the concept of “neighborhood environment” and to examine the types of neighborhood environmental factor(s) that may have a significant relationship with youth marijuana use. When adolescents live in low-income, high-crime, drug-dealing, trash-ridden neighborhoods, would any of these neighborhood characteristics matter more than others in predicting marijuana use? Indeed, there is growing evidence that neighborhood disadvantage may contribute to substance using behaviors among adults and youth [5,6,7,8,9,10]. A recent prospective study of neighborhood characteristics in the United States found that living in a neighborhood with higher unemployment was associated with a higher incidence of adolescents initiating marijuana use and binge drinking [10]. Research conducted in Chile has found neighborhood disadvantage to be associated with perceptions of neighborhood safety [4,11]. A recent study found that living in neighborhoods with greater access to recreational spaces is inversely associated with cigarette smoking among adolescents [12]. In another study of neighborhood effects in Chile, it was found that financial stress was predictive of a greater probability of domestic violence but the effect was larger among residents living in neighborhoods with greater amount of trash in their streets [13]. It is plausible that neighborhoods with greater unemployment rates, more trash, and drug availability act as chronic stressors [14] that influence residents’ mental health and drug using behaviors. 

Identification of specific factors that may be related to youth marijuana use requires urgent attention considering the high prevalence of marijuana use in Chile and the possibility of de-criminalizing marijuana use among adults in Chile and the recent legalization of marijuana use in Uruguay. It is within this context that in 2011, the most recent national survey of drug use among school-attending youth in Chile, estimated that 26.5% of 8th through 12th graders had smoked marijuana in their lives (14.4% for 8th graders and 37.1% for 12th graders) and that approximately 11.2% had used marijuana in the past month (9.9% of 8th graders and 12.9% of 12th graders) with minimal differences between adolescent males and females but with greater percentages among adolescents attending public schools than private schools highlighting socioeconomic differences [15]. The large percent of adolescents consuming marijuana is of concern especially in light of the research showing the negative effects of this drug on adolescent development [16,17]. For example, a study with Chilean adolescents found marijuana to negatively affect their cognitive functioning, which can in turn lead to poor academic performance [18]. 

In this study we report findings of a prospective study of neighborhood characteristics and marijuana use among adolescents in Santiago, Chile. We disaggregated the concept of “neighborhood environment” into three distinct measures. First, considering that variations in drug availability at the neighborhood level have been associated with variations in opportunities to use drugs and hence actual drug use [6,19,20], this study examined adolescents’ reports of the selling and use of drugs in their neighborhoods. In addition, we considered adolescents’ reports of other aspects of neighborhood delinquency and crime (e.g., problems with muggings, burglaries, and assaults in their neighborhoods). Lastly, we also assessed characteristics of the built-environment (e.g., abandoned buildings, drug paraphernalia in the streets, traffic noise, strong odors due to urine or feces, trash, street dogs) based on Systematic Neighborhood Observations [21] of the immediate area where youth live. These observations are important not only to further understand the environments in which adolescents live but also because Santiago residents have proposed changes in the built-environment as strategies to improve neighborhood safety [3,22]. We also tested if the potential associations between neighborhood disadvantage and marijuana use varied by sex as it is possible that adolescent females experience neighborhood stressors differently than adolescent males resulting in different outcomes for the youth.

This study is timely as the Chilean government has taken great interest in reducing the prevalence of illicit drug use [23] and to increase neighborhood-level efforts to reduce crime, including the selling of illicit drugs [24]. The results of this research can inform neighborhood-level programs and interventions to reduce marijuana use among adolescents in Santiago, Chile.

## 2. Methods

### 2.1. Sample

Data for this study are from adolescents participating in the Santiago Longitudinal Study (SLS), a study of urban adolescents in Santiago, Chile. The SLS is a collaborative project between Chilean and U.S. institutions with funding from the National Institute on Drug Abuse (NIDA). Participants for this study included 1,076 youth (51.6% male) who participated in a study of substance using behaviors from December 2007 to 2010 when the youth were 12–17 years old (Time 1). A total of 771 adolescents (71.7%) were successfully re-interviewed when they were 14–19 years old in 2008–2011 (Time 2). Study participants came from a community based sample of low SES families from working-class “comunas” (municipalities) in the southern part of the city of Santiago who had earlier participated in a study of nutrition [25]. Several of these “comunas” face considerable public health challenges with drug trafficking, selling, and use within their communities [4]. The analytic sample consisted of the 725 adolescents with complete data on all of the variables at Time 1 and Time 2. 

At Time 1, adolescents completed a two-hour interviewer-administered questionnaire to assess constructs measured in the study such as substance using behaviors, perceptions of neighborhood crime, drug use and selling, behavioral problems, and health, among others. At Time 2, the adolescents also completed an interviewer-administered questionnaire but due to funding constraints this was a shortened version of the questionnaire administered at Time 1. At Time 2, the questionnaire focused mainly on substance use questions. Interviews were conducted in Spanish in a private office at the study site by Chilean psychologists trained in the administration of instruments. Interviewers obtained adolescent assent and parental consent prior to commencing the interviews. The study received Institutional Review Board approval from the institutional review boards of the corresponding universities. 

In addition, with supplemental funding from NIDA, we conducted a Systematic Neighborhood Observation (SNO) in 2008–2010. The SNO consisted of a systematic assessment of the built-in environment of the immediate area where youth lived. Social workers used a 21-item instrument to rate the amount of greenery, garbage, street dogs, drug paraphernalia, street noise and traffic volume, graffiti, discarded cigarettes and alcohol cans, overall trash, quality of housing exteriors, and whether people were congregating on sidewalks or in the street, among other items. Social workers conducting the assessment walked counterclockwise around the neighborhood blocks, rating each “cuadra” (a block face) until the “manzana” (neighborhood block) where the youth live was rated. Approximately 3,600 “cuadras” consisting of approximately 900 “manzanas” were rated. The SNO was conducted at Time 1.

### 2.2. Measures

#### 2.2.1. Dependent Variables

The dependent variable, measured at Time 2 (approximately a year and a half after Time 1), was the number of occasions the adolescent had smoked marijuana in the past year prior to the interview with response categories “0 = no”, “1 = 1–2 occasions”, “2 = 3–5 occasions”, “3 = 6–9 occasions”, “4 = 10–19 occasions”, “5 = 20–39 occasions”, and “6 = 40 or more occasions”. For completeness, adolescents who indicated they had never smoked marijuana or had smoked but not in the past 12 months were coded as zero. 

#### 2.2.2. Independent Variables—Neighborhood Characteristics

There were three variables measuring neighborhood characteristics, all assessed at Time 1. The first two variables were based on adolescents’ reports, while the third was based on the SNO. The first variable, *neighborhood crime*, was based on the question that asked adolescents: How often are there problems with muggings, burglaries, assaults or anything else like that in your neighborhood? Would you say “1 = never”, “2 = hardly ever”, “3 = not too often”, “4 = fairly often” and “5 = very often”. The second variable, *neighborhood drug selling/use*, was based on the combined mean scores of the questions that asked adolescents: How much of a problem is the selling and use of drugs in your neighborhood? Would you say this problem is “1 = never”, “2 = not serious at all”, “3 = not too serious”, “4 = fairly serious”, “5 = very serious”, and: During the past 12 months, how often have you seen people selling illegal drugs in your neighborhood? With response categories “1 = never”, “2 = a few times a year”, “3 = once or twice a month”, “4 = at least once a week”, and “5 = almost every day”. The inter-item reliability (Cronbach’s alpha) for this variable was 0.65. The third variable was an *index of noxious neighborhood characteristics* created by summing the social workers’ ratings of the neighborhood blocks. Ratings consisted of Yes/No response categories to items about quality of the built environment such as traffic noise, strong odors (due to urine, feces, alcohol, or trash), trash (e.g., broken glass, paper, cans of foods), beer containers or liquor bottles on the street, extent of graffiti, cigarette butts, street dogs, and so on. Because this variable is considered an index and not an underlying construct, inter-item reliability was not computed. 

#### 2.2.3. Demographic and Control Variables

Demographic characteristics assessed included the adolescents’ self-reported age and sex and their family’s SES, also measured at Time 1. An SES index, a z-transformed scale, was created that consisted of a weighted linear combination of four items: mother’s and father’s completed years of education, monthly family income, and the higher of the occupational prestige scores of the mother or father. The SES index demonstrated high reliability (α = 0.81). The variable occasions of marijuana use in the past year assessed at Time 1, measured in the same manner as the dependent variable (measured at Time 2), was included as control.

### 2.3. Analysis

Data were analyzed with bivariate statistics and with ordinal logistic regression, and the Brant test [26] was used to test the proportional odds assumption with Stata 13.0 [27]. Demographics and occasions of marijuana use in the past year assessed at Time 1were included as controls. Also tested were interactions between the neighborhood and sex variables. 

## 3. Results

At Time 1, 6.6% of adolescents had ever used marijuana whereas at Time 2, 26.8% had ever used marijuana (see Table 1).

As shown in Table 2, the corresponding percentage of adolescents who had used marijuana one to two times in the past year at Times 1 and 2 are 3.7% and 8.1%, respectively. At Time 1 less than 1% had used marijuana more than 20 times in the past year; by Time 2 the number had risen to nearly 3%. 

As shown in Table 3, results of the ordinal logistic regression indicate that as the neighborhood levels of drug availability at Time 1 increased, but not crime or the noxious environment, adolescents had higher odds of occasions of marijuana use at Time 2 (odds ratio [OR] = 1.39; 95% CI = 1.16–1.66), controlling for age, sex, SES, and occasions of marijuana use at Time 1 (see Table 3). The Brant test indicated the proportional odds assumption was not violated. Age (OR = 1.51; 95% CI = 1.22–1.85) and prior (Time 1) occasions of marijuana use (OR = 1.78; 95% CI = 1.31–2.42) were also positively associated with occasions of marijuana use at Time 2. The odds of occasions of marijuana use were lower for adolescent females than males (OR = 0.64; 95% CI = 0.43–0.95). As none of the interactions were statistically significant these were omitted from the final analysis. 

**Table 1:** Descriptive characteristics of the study participants (*N* = 725).

Variables	Mean (sdev)	%	Range
Time 1			
Age	13.8 (1.0)		11.9–16.9
Sex (Female)		50.0	
SES (z-score)	0.0 (1.0)		−4.1–4.7
Neighborhood crime	2.8 (1.2)		1–5
Neighborhood drug selling	2.9 (1.2)		1–5
Noxious neighborhood	8.7 (1.4)		5.3–16.3
Ever used marijuana		6.6	
Time 2			
Age	16.0 (1.3)		13.0–19.6
Ever used marijuana		26.8	

**Table 2:** Percent of adolescents by occasions of marijuana use in the past year at Times 1 and 2 (*N* = 725).

Past year occasions of marijuana use	Time 1		Time 2	
	*n*	%	*n*	%
Never used or not in the past year	686	94.6	591	81.5
1–2	27	3.7	59	8.1
3–5	4	0.5	22	3.0
6–9	3	0.4	14	2.0
10–19	1	0.2	18	2.5
20–39	1	0.2	7	1.0
40 or more	3	0.4	14	1.9

**Table 3:** Prospective association of neighborhood characteristics and occasions of marijuana use: Results of ordinal logistic regression (*N* = 725).

Predictors (all T1 variables)	Odds Ratio	95% CI
Independent		
Neighborhood crime	0.91	0.75–1.10
Neighborhood drug selling/use	1.39	1.16–1.66
Noxious neighborhood	1.03	0.90–1.19
Controls		
Age	1.51	1.22–1.85
Sex (Ref = Male)	0.64	0.43–0.95
SES	1.08	0.88–1.30
Occasions of marijuana use	1.78	1.31–2.42

## 4. Discussion

In this study we examined the relationship of neighborhood characteristics with marijuana use among a community based sample of adolescents living in families and neighborhoods of low SES and where the presence of drugs is common [22]. We found that living in “poverty” was not prospectively associated with adolescent marijuana use approximately a year and a half to two years later. The variables “general” crime (e.g., burglaries, muggings) and noxious neighborhood environment were not identified as significant factors associated with marijuana use. Rather, it was exposure to drugs (presence of dealers and people using drugs) in their neighborhoods that predicted occasions of marijuana use. These findings suggest that “poverty”, “crime”, and “presence of drugs in the neighborhood”, though related, are sufficiently different phenomena to differentially impact substance using behaviors, or at least marijuana use among a Chilean sample. 

For policy development, the findings of the current study suggest that broad based substance abuse prevention efforts to “improve the community”, though important for the residents’ overall quality of life, may result in little effect on subsequent adolescent drug use if opportunities to use remain accessible. Instead, the study findings suggest that attempts to reduce adolescent drug use in low income neighborhoods may do best if efforts concentrated on specific features of the “substance abuse environment” of those neighborhoods. For example, communities may organize themselves to prevent drug houses from becoming established, work with the police to minimize or eliminate drug trafficking and selling in the neighborhoods particularly when this occurs during the day when minors may be walking to and from school or playing outside after school, prevent drug dealing and drug use from occurring in and around their local school grounds, assist parents improve the monitoring of their adolescents, and increase supervised after-school activities through participation in clubs and sports. 

The study findings should be interpreted with the following limitations in mind. First, the SNO were based on three social workers who independently rated the neighborhood blocks. Although everyone received the same training and supervision, budgetary constraints prevented us from assessing inter-observer reliability. Second, data from the Chilean Census are not available at the neighborhood block level and although some data are available at the municipality level, these data are not available for all 27 “comunas” (or counties) represented in this study and therefore these data were not included in the study. Finally, the sample of adolescents is based on a convenience sample from neighborhoods of mid to low SES and thus it is not clear the extent to which the findings can be generalized to the larger population of Chilean youth. Notwithstanding these limitations, the current study offers many strengths as well as valuable insights for further research. Strengths of this study include the large sample of community-dwelling adolescents from a developing country, the prospective examination of the associations between neighborhood characteristics and marijuana use, the use of both self-reported neighborhood characteristics that are disaggregated into two constructs, and what we believe the first ever systematic observation of neighborhoods in a Latin American country. 

Future research with other populations and neighborhood contexts is needed to corroborate the findings of the current study. More specifically, more research is needed to better understand the process by which adolescents are influenced to use, or refrain from using, marijuana when their neighborhood contexts are such that the opportunities to use marijuana and other illicit drugs are a constant threat, potentially normative behavior, net of other neighborhood characteristics, adolescent personality factors, and family influences. Although it is certainly important to increase our understanding of the mechanisms by which neighborhood-level drug dealing and use influence adolescent marijuana use, it is just as important to learn of the strategies that adolescents utilize to remain drug free particularly among those who live in environments where illicit drugs are easily available. That is, more research is needed with urban youth worldwide to further understand the processes by which youth cognitive and behavioral decision-making, peer influences, parental monitoring, and contextual influences (e.g., interactions among community members and law enforcement, national and local drug policies) influence some, but not all or the majority of youth to use marijuana. 

## 5. Conclusions

The findings suggest that “poverty”, “crime”, and “drug problems” may not be synonyms and thus can be understood discretely. As Latin American countries re-examine their drug policies, especially those concerning decriminalizing marijuana use, the findings suggest that attempts to reduce adolescent marijuana use in disadvantaged neighborhoods may do best if efforts are concentrated on specific features of the “substance abuse environment”.
